# Two-hour glucose reductions and baseline levels for effective prevention of type 2 diabetes in individuals with impaired glucose tolerance: An evidence synthesis and meta-regression analysis

**DOI:** 10.1016/j.pmedr.2026.103571

**Published:** 2026-07-13

**Authors:** Kotoba Okuyama, Anna Tsutsui, Yoshitaka Murakami

**Affiliations:** a Department of Medical Statistics, Toho University Graduate School of Medicine, 5-21-16 Omori-nishi, Ota-ku, Tokyo, Japan; b Department of Medical Statistics, Faculty of Medicine, Toho University, 5-21-16 Omori-nishi, Ota-ku, Tokyo, Japan

**Keywords:** Impaired glucose tolerance, Type 2 diabetes mellitus, Prevention, Meta-regression analysis, Risk reduction

## Abstract

**Objective:**

To identify the optimal 2-h glucose reduction and baseline level for preventing progression from impaired glucose tolerance to type 2 diabetes using meta-regression.

**Methods:**

We systematically reviewed randomized clinical trials of lifestyle modifications and/or anti-hyperglycemic medications reporting both 2-h glucose and diabetes prevention in impaired glucose tolerance. PubMed (1997), Cochrane Library (1995), and Ichushi-Web (Japanese medical literature database; 2000) were searched from inception through January 26, 2025. A meta-regression model examined log-transformed hazard ratios for diabetes progression, with 2-h glucose reduction and baseline level as predictors.

**Results:**

From 1372 candidates, 32 studies (*n* = 33,839 participants) were analyzed. The model indicated linear relationship and the early intervention at a baseline 2-h glucose of 140 mg/dL (7.8 mmol/L) could prevent 44% of diabetes with only a 10 mg/dL (0.56 mmol/L) reduction. Conversely, later interventions at a baseline 2-h glucose of 170–190 mg/dL (9.4–10.6 mmol/L) require larger 2-h glucose reductions of 20–30 mg/dL (1.1–1.7 mmol/L) for similar preventive effects (40–42%); which may necessitate pharmacotherapy.

**Conclusions:**

This meta-regression analysis demonstrates that early and minimal interventions for impaired glucose tolerance are optimal, suggesting that its timely detection is vital for diabetes prevention.

## Highlights

• Meta-regression of type 2 diabetes prevention in impaired glucose tolerance.
• Reductions in 2-h plasma glucose and their baseline levels were evaluated.
• Lifestyle modification is most effective in early-stage impaired glucose tolerance.
• Higher baseline 2-h plasma glucose may warrant pharmacological therapy.

## 1 Introduction

The global burden of diabetes is expected to rise from 589 million cases in 2024 to 853 million cases by 2050, with rapid increases in newly industrialized Asian countries such as China and India (Duncan et al., 2025). Given that impaired glucose tolerance progresses to diabetes more easily than impaired fasting glucose (Rao et al., 2004; Richter et al., 2018), screening for impaired glucose tolerance may help to identify at-risk group that could benefit from preventive interventions. However, the effectiveness of such interventions may depend on the timing of their introduction relative to impaired glucose tolerance severity. For example, conventional non-pharmacological interventions, such as diet and exercise, may be effective for individuals with early-stage impaired glucose tolerance. In contrast, the late-stage impaired glucose tolerance often present with significant pathophysiological abnormalities that require more intensive interventions (including pharmacotherapy) to halt the progression of dysglycemia. Therefore, studies have reported that early intervention is preferable due to the difficulties in reversing later-stage impaired glucose tolerance (Tuomilehto and Schwarz, 2016; An et al., 2024). While various interventions have been employed to prevent diabetes in impaired glucose tolerance, no single study has consolidated the existing evidence to determine their optimal 2-h glucose reduction and baseline level.

This study aims to clarify the optimal 2-h glucose reduction and baseline level across different impaired glucose tolerance stages based on data from randomized clinical trials. Herein, we employed a meta-regression approach to quantify the relationships between intervention-induced changes on postprandial glucose and diabetes prevention rates in individuals with impaired glucose tolerance.

## 2 Methods

In this study, we constructed meta-regression models using aggregate data from multiple randomized clinical trials to examine the relationships between 2-h glucose —plasma glucose measured 2 hour after a 75-g oral glucose tolerance test— and type 2 diabetes prevention in impaired glucose tolerance. Eligible randomized clinical trials were identified through a systematic review.

The systematic review protocol was registered in the University Hospital Medical Information Network Clinical Trials Registry (ID: UMIN000055889). This review was conducted in accordance with PRISMA 2020 guidelines (Page et al., 2026). A systematic search was performed across PubMed (1997), the Cochrane Central Register of Controlled Trials (Cochran Library; 1995), and Ichushi-Web (a Japanese bibliographic database of medical journals; 2000) from the earliest available dates until January 26, 2025. The search strategies were developed in collaboration with a medical librarian from the Toho University Media Center, and are presented in the Supplementary Materials (**Supplementary Tables S1-S3**).

We included studies that met the following criteria: (i) comprised prediabetes identified using 2-h glucose alone or in combination with fasting glucose, (ii) randomized clinical trial, (iii) reported number of new-onset diabetes, excluding the post-intervention period, (iv) interventions involved lifestyle modifications and/or anti-hyperglycemic agents, (v) minimum intervention duration of 6 months, (vi) reported both the baseline and post-intervention 2-h glucose, (vii) full text accessible, and (viii) published in English or Japanese. The exclusion criteria were as follows: (i) included isolated impaired fasting glucose alone or non-prediabetes, diabetes, or normal glucose tolerance; and (ii) children aged ≤18 years, gestational diabetes, or other diseases.

All search results were imported into the Rayyan platform (Rayyan Systems Inc., Cambridge, MA, USA) for screening. Article titles and abstracts were independently screened for relevance by two reviewers (KO and AT), and all discrepancies were resolved through mutual discussion.

The two reviewers independently extracted data from the full text of the selected articles. The data items included publication details (study name/first author, publication year) and study characteristics (intervention[s], number of participants, duration of intervention, outcome[s], and covariates). All discrepancies were resolved through mutual discussion.

The study outcome was the hazard ratio for diabetes progression (“event”). When hazard ratio was not reported, we assumed that the survival function in the intervention and control groups followed an exponential distribution: $S\left(t\right)=\exp\left(-\mathrm{\lambda t}\right)$, where λ is the hazard rate during the intervention duration and t is the median or mean intervention duration. Therefore, λ was calculated as $-\ln\left[S\left(t\right)\right]/t$. The cumulative event rate at t, denoted as $F\left(t\right)\left[=1-S\left(t\right)\right]$, was estimated based on the number of events at t divided by the number of participants in each group, and the group-specific hazard rate was then calculated using the formula defined above. The hazard ratio was calculated as the ratio of hazard rates between the intervention and control groups. The durations of studies included in the meta-analysis were all less than 6 years. This relatively comparable follow-up periods across studies did not necessitate adjustment for this outcome. The hazard ratios reported in the articles and those calculated from the number of events were generally consistent (**Supplementary Fig. S1**). We also extracted the group-specific event counts to determine each study's statistical weight in the meta-regression models. Studies without any events in one or both groups were excluded from analysis.

We hypothesized that, regardless of the intervention type, any strategy reducing postprandial glucose would favorably impact the prevention of diabetes. To represent postprandial glucose, we utilized 2-h glucose which is among the strongest predictors of diabetes progression (Noble et al., 2011; Nielsen et al., 2016). Consequently, we evaluated the relationship between the hazard ratio for diabetes progression and the between-group difference in 2-h glucose reduction from baseline (intervention vs. non-intervention) to elucidate the association between postprandial glucose control and its preventive efficacy. The 2-h glucose reduction was measured at the earliest available post-intervention time point, as this was considered to best reflect the true intensity of each intervention. In the included studies, 2-h glucose reductions were generally consistent over the intervention durations. Fasting glucose reductions were also determined in the same manner, but hemoglobin A1c reductions were not analyzed because they were rarely available. The other candidate predictors were the weighted mean values of baseline 2-h glucose, fasting glucose, hemoglobin A1c, and body mass index. These were weighted according to the number of participants in each group, and used to estimate the mean values in the total population of the study. Body mass index, rather than body weight, was selected as the anthropometric measure of obesity. Age was not examined in this analysis.

To evaluate the relationships between 2-h glucose reduction and the hazard ratios for diabetes progression, we constructed meta-regression models using weighted linear regression (Borenstein et al., 2009; Schmid et al., 2020). The response variable was the natural log-transformed hazard ratio because it approximates a normal distribution. The weight for each study was calculated as the inverse variance of log-transformed hazard ratio (Tierney et al., 2007; Perneger, 2008). First, a univariate meta-regression model was constructed with only 2-h glucose reduction as the independent variable of interest. Next, multivariable meta-regression models were constructed for all combinations of predictors, with 2-h glucose reduction retained in all models. Before conducting the multivariable analyses, we examined the correlation coefficients (r) for all pairs of predictors. To prevent multicollinearity, we excluded one predictor from any pair with a correlation coefficient exceeding 0.8. Candidate models were constructed using predictors that met a significance threshold of *p* < 0.2. These models were systematically compared to select the final meta-regression model using adjusted *R*^2^ values to assess goodness-of-fit. From the final meta-regression model, we estimated the risk reductions across 24 hypothetical scenarios defined by combinations of six levels of baseline 2-h glucose and four levels of 2-h glucose reduction. The baseline 2-h glucose were shown as 140, 150, 160, 170, 180, and 190 mg/dL (7.8, 8.3, 8.9, 9.4, 10.0, and 10.6 mmol/L, respectively); the lowest two values represented early-stage impaired glucose tolerance, the middle two values represented middle-stage impaired glucose tolerance, and the highest two values represented late-stage impaired glucose tolerance. Next, the 2-h glucose reduction levels were shown as −10, −20, −30 and −40 mg/dL (−0.56, −1.1, −1.7 and −2.2 mmol/L, respectively). We first predicted the hazard ratios under the 24 hypothetical scenarios, and subsequently calculated the corresponding risk reductions (%) for diabetes progression using the formula (1− hazard ratio) × 100. To exploratorily evaluate whether fasting glucose parameters could achieve comparable predictive performance to 2-h glucose parameters, a parallel meta-regression analysis was performed using fasting glucose reduction and baseline fasting glucose. Finally, a leave-one-out sensitivity analysis was conducted by iteratively reconstructing the meta-regression models and sequentially omitting one study at a time to assess its influence on the overall results. All analyses were performed using SAS release 9.40 (SAS Institute Inc., Cary, NC, USA), and *p*-values <0.05 were considered significant.

The study protocol was submitted to the Ethics Committee of the Faculty of Medicine, Toho University (A24005, April 3, 2024), which waived the need for formal ethical approval or informed consent due to the use of aggregate data from previously published studies in accordance with institutional guidelines for the protection of human subjects, including safety and privacy.

## 3 Results

**Fig. 1:**
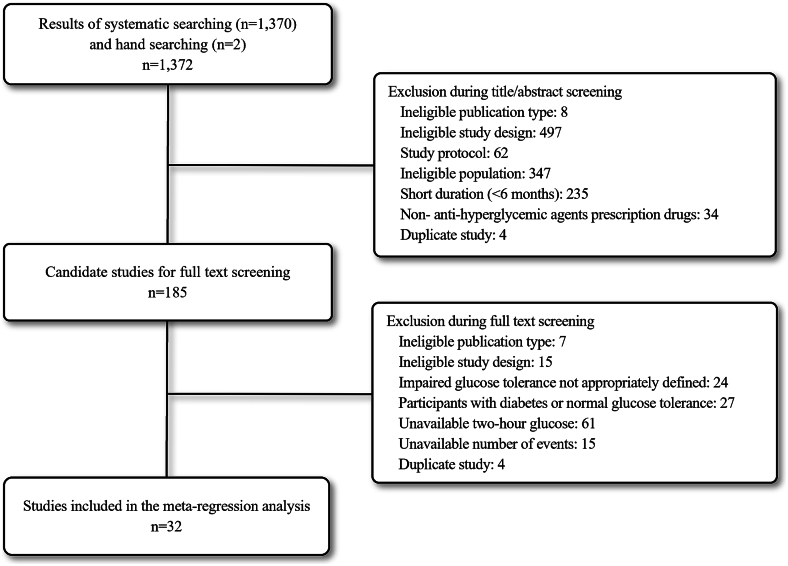
Flow diagram of study selection for the systematic literature search on type 2 diabetes prevention trials in adults with impaired glucose tolerance reporting 2-h glucose data (1984–2024).

We identified 1372 candidate articles through systematic searching (*n* = 1370) and hand searching (*n* = 2). After screening the titles and abstracts, we retrieved the full texts of 185 articles, of which 32 were determined to be eligible for inclusion (Fig. 1). The bibliographic information for the included studies is provided in **Supplementary Table S4**. The 32 studies encompassed a total of 33,839 participants and 6983 diabetes progression events. The study characteristics are summarized in Table 1. The lifestyle modification-based interventions included diet, exercise (e.g., yoga), Short Message Services to disseminate diabetes-related information, and use of Chinese medicines and/or supplements (e.g., *jinlida*, *tangzhiping*, vitamin D, calcium, zinc, protein, L-arginine, fiber, fenugreek, and herb mix). The pharmacotherapy-based interventions included anti-hyperglycemic agents such as alpha-glucosidase inhibitors, biguanides (metformin), dipeptidyl peptidase-4 inhibitors, glucagon-like peptide-1 receptor agonists, glinides, sulfonylureas, and thiazolidinediones. The magnitude of 2-h glucose reduction varied substantially across interventions. While some lifestyle modifications induced approximate reductions of 10 mg/dL (0.56 mmol/L), other lifestyle modifications resulted in larger reductions of 40 mg/dL (2.2 mmol/L). Pharmacotherapies indicated approximate reductions of 20–30 mg/dL (1.1–1.7 mmol/L).

**Table 1:** Characteristics of randomized controlled trials included in the meta-regression analysis: studies of type 2 diabetes prevention in adults with impaired glucose tolerance reporting 2-hour glucose data (1997–2024 Systematic Search).

Study ID and study name/first author (publication year)	Intervention	Median or mean study duration (years)	Intervention n/N	Control n/N	Relative effect (vs. control)			Mean/median baseline e in the study population				
					Hazard Ratio^c^	Two-hour glucose [Change from baseline] (mg/dL)	Fasting glucose [Change from baseline] (mg/dL)	Two-hour glucose (mg/dL)	Fasting glucose (mg/dL)	Hemoglobin A1c (%)	Body Mass Index (kg/m^2^)	Age (years)
1. Da Qing DPS (1997)	Lifestyle modification a	6.0 b	58/ 126	90/ 135	0.58 c	−41.6	−10.6	162.0	100.6	NA	25.8	45.0
2. Li (1999)	Metformin	1.0 b	3/ 42	6/ 43	0.49 c	−5.4	−10.8	162.8	128.0	7.4	26.2	49.5
3. Finnish DPS (2001)	Lifestyle modification	3.2	27/ 265	59/ 257	0.40	−10.0 d	−5.0 d	159.0	109.0	NA	31.2	55.0
4. Oldroyd (2006)	Lifestyle modification	2.0 b	7/ 37	8/ 32	0.73 c	−0.9 d	−2.3 d	165.3	109.8	NA	NA	57.9
5. Eriksson (2006)	Glipizide	1.5 b	1/ 17	5/ 17	0.20 c	−4.1 d	−1.3 d	144.9	95.4	NA	28.4	56.5
6. DREAM (2006)	Rosiglitazone (± ramipril)	3.0	231/ 2635	555/ 2634	0.38	−28.8 d	−9.0 d	156.6	104.4	NA	30.9	54.7
7. DAISI (2008)	Acarbose	3.0 b	11/ 60	14/ 58	0.76	−21.1	−5.04	171.9	117.9	5.8	28.9	57.5
8. IDPP-1 (2009)	Lifestyle modification (± metformin) a	2.5	132/ 321	62/ 116	0.69 c	−16.2	−5.4	153.5	97.7	6.2	25.6	NA
9. NAVIGATOR (2010)	Nateglinide (± valsartan)	5.0	1674/ 4645	1580/ 4661	1.07	4.4 d	0.47 d	165.6	109.8	5.8	30.5	63.8
10. ACT NOW (2011)	Pioglitazone	2.4	15/ 303	50/ 299	0.28	−14.9 d	−3.6 d	168.0	105.0	5.5	33.7	52.3
11. SLIM (2011)	Lifestyle modification (+ metformin)	4.1	22/ 74	41/ 73	0.53 c	−10.8	−1.3	158.9	107.4	5.9	29.8	56.9
12. JDPP (2011)	Lifestyle modification	3.0 b	9/ 146	18/ 150	0.47 c	−9.0	1.8	163.7	108.1	NA	24.6	51.0
13. Saito (2011)	Lifestyle modification (frequent intervention)	2.7	35/ 311	51/ 330	0.56	−8.0 d	−2.0 d	134.0	107.5	5.4	27.0	49.0
14. Moore (2011)	Lifestyle modification	0.5 b	27/ 208	7/ 99	1.92 c	−10.4	−3.4	149.7	105.9	NA	29.7	61.3
15. Monti (2012)	Supplement (L-arginine)	1.5 b	15/ 72	15/ 72	1.04	−5.29 d	2.79 d	162.4	104.8	5.9	30.0	57.7
16. Gao (2013)	Chinese medicine (tangzhiping)	3.0 b	51/ 255	100/ 255	0.51 c	−40.1	−19.8	168.9	104.2	5.7	25.2	50.2
17. Wong (2013)	Lifestyle modification (short message service)	2.0 b	6/ 54	9/ 50	0.62	−12.6	−1.6	133.2	105.8	NA	25.9	54.6
18. Dutta (2014)	Vitamin D + cholecalciferol (+ calcium) a	2.4	6/ 68	13/ 57	0.37	−20.3	−6.2	154.4	109.8	6.1	26.3	47.6
19. Gaddam (2015)	Supplement (fenugreek)	3.0 b	17/ 74	36/ 66	0.40 c	−15.1	−1.8	144.4	103.3	NA	26.3	NA
20. Jorde (2016)	Supplement (vitamin D)	5.0 b	103/ 256	112/ 255	0.90	0.18 d	−1.44 d	132.0	109.5	6.0	30.0	62.1
21. ACE (2017)	Acarbose	4.4	436/ 3272	513/ 3250	0.82	−5.4 d	NA	167.4	99.0	5.9	25.4	64.3
22. Roux (2017)	Liraglutide	3.1 b	26/ 1505	46/ 749	0.21	−25.2 d	−7.6 d	133.2	99.0	5.8	38.9	47.4
23. Hu (2017)	Lifestyle modification	1.0 b	9/ 214	43/ 220	0.20	−3.3 d	−6.1 d	155.2	110.4	5.8	23.7	69.4
24. OptiFiT (2018)	Supplement (fiber)	2.0 b	9/ 89	16/ 91	0.55 c	−5.8 d	NA	163.8	107.1	5.6	32.4	59.5
25. Ranasinghe (2018)	Supplement (zinc)	1.0 b	11/ 100	25/ 100	0.37 c	−28.1	−20.1	164.6	114.9	NA	25.1	51.8
26. Niroomand (2019)	Supplement (vitamin D_3_)	0.5 b	1/ 81	4/ 81	0.25 c	−3.0 d	1.0 d	140.5	107.5	NA	31.5	46.5
27. Nakanekar (2019)	Supplement (herb mix)	0.5 b	8/ 57	15/ 57	0.53 c	−21.4	−8.1	163.8	108.2	6.4	28.3	48.0
28. PRELLIM (2020)	Linagliptin (+ metformin)	1.5	4/ 74	10/ 70	0.25	−19.0	−7.0	165.5	105.1	5.5	30.7	49.0
29. PREVIEW (2020)	High protein (± high-intensity physical activity) a	3.0 b	30/ 1111	32/ 1112	0.82	1.0 d	NA	138.1	111.6	5.6	35.4	51.5
30. PREVENT-WIN (2020)	Supplement (vitamin D) (+ calcium)	1.5 b	1/ 61	3/ 60	0.48 c	4.2	−1.4	127.1	111.7	NA	30.0	NA
31. FOCUS (2024)	Chinese medicine (jinlida)	2.2	123/ 442	189/ 443	0.59	−9.2 d	−3.8 d	164.7	105.6	5.9	26.8	52.6
32. IPDS (2024)	Yoga (+ lifestyle modification)	3.0 b	56/ 488	92/ 486	0.57	0.02	2.0	152.1	105.5	5.8	28.8	44.1

N refers to the total number of participants, while n refers to the number of participants who progressed to type 2 diabetes. Two-hour glucose was measured glucose at two hours after a 75-g oral glucose tolerance test.

a If a study used two or more interventions, the following approaches were used to avoid correlations between any two observations: One (diet + exercise) group from three interventions (diet only, exercise only, and diet + exercise) was selected [Study #1]; Only pooled data from three interventions (lifestyle modification, metformin, and lifestyle modification + metformin) was used [Study #8]; A reference group with a different population was excluded [Study #18]; Only pooled data from two interventions and two controls were selected for the intervention and control, respectively [Study #29].

b If the median or mean durations were not reported, the total study duration was utilized [Study #1, #2, #4, #5, #7, #12, #14, #15, #16, #17, #19, #20, #22, #23, #24, #25, #26, #27, #29, #30 and #31].

c If hazard ratios were not reported, the hazard rates of the intervention and control groups were estimated based on the cumulative event rate of type 2 diabetes progression and the study duration in each group under the assumption that the group-specific survival functions followed an exponential distribution. Next, the hazard ratio was calculated as the ratio of hazard rates between the intervention and control groups [Studies #2, #4, #8, #14, #24, #26 and #30]. If risk reductions were reported, hazard ratios were estimated as 1 minus risk reduction [Studies #1, #5, #11, #12, #16, #19 and #27]. If odds ratios were reported, they were used as approximations of hazard ratios [Study #25].

d If neither the change from baselines of the two groups nor their differences were reported, the change from baseline of each group was calculated by subtracting the baseline value from the post-intervention value; next, the inter-group differences in change from baselines were calculated [Studies #3, #4, #5, #6, #9, #10, #13, #15, #20, #21, #22, #23, #24, #26 and #31]. One study reported only change from baselines for two subgroups in each group; the weighted average of the change from baselines from the two subgroups were calculated for each group [Study #29]. Glucose measurements (i.e., 2-h glucose and fasting glucose) reported in mmol/L were converted to mg/dL by multiplying the values by 18.

e Mean baseline values in the study population were calculated as the weighted means from the two groups. The number of participants in each group served as the weighting factor.

**Fig. 2:**
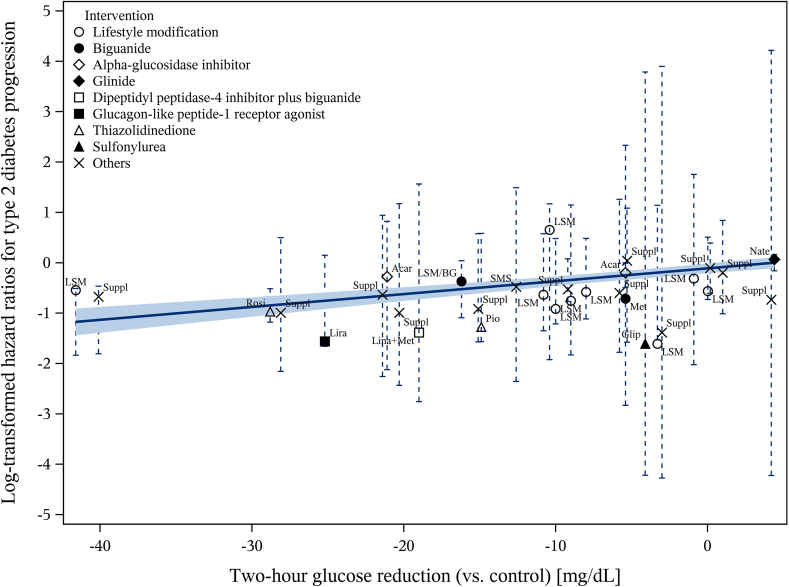
Univariate meta-regression of log hazard ratios for type 2 diabetes progression by 2-hour glucose reduction (vs. control) in type 2 diabetes prevention trials of adults with impaired glucose tolerance reporting 2-hour glucose data (1997–2024 Systematic Search). The solid blue line indicates the predicted meta-regression line, and the shaded blue area represents its 95% confidence band. The dashed vertical lines indicate the predicted 95% confidence intervals for individual predicted values in each study. “Other” refers to Chinese medicines, supplements (e.g., vitamin D and calcium), and SMS. Abbreviations: Acar, acarbose; AGI, alpha-glucosidase inhibitor; BG, biguanide; DPP4, dipeptidyl peptidase-4 inhibitor; Glip, glipizide; GLP-1, glucagon-like peptide-1 receptor agonist; Lina, linagliptin; Lira, liraglutide; LSM, lifestyle modification; Met, metformin; Nate, nateglinide; Pio, pioglitazone; Rosi, rosiglitazone; SMS, Short Message Service; SU, sulfonylurea; Suppl, supplements; TZD, thiazolidinedione. (For interpretation of the references to colour in this figure legend, the reader is referred to the web version of this article.)

Fig. 2 shows the log-transformed hazard ratio for diabetes progression plotted against differences in 2-h glucose reduction relative to the control group. In the univariate meta-regression model, 2-h glucose reduction was significantly associated with the log-transformed hazard ratio values (*p* < 0.001). The prediction formula based on this model is as follows, with an adjusted *R*^2^ of 64%:

(1) $$\ln\left(Hazard\;Ratio\right)=-0.11174+0.02554\times 2\;hour\;glucose\;\mathrm{reduction}\;\left[\mathrm{mg}/\mathrm{dL}\right]$$

Before the multivariable analyses, fasting glucose reduction was excluded from the model because it was highly correlated with 2-h glucose reduction (*r* = 0.932). Mean baseline hemoglobin A1c was also excluded because it was not reported in 12 of the 32 studies (37.5%). All combinations of mean baseline 2-h glucose, fasting glucose, and body mass index were sequentially evaluated as predictors in the meta-regression models in addition to 2-h glucose reduction (**Supplementary Table S5**). We identified two candidate models that achieved (or marginally achieved) *p* < 0.2 for all included predictors. The first model included baseline 2-h glucose (*p* = 0.041) and 2-h glucose reduction (*p* < 0.001), while the second model included baseline body mass index (*p* = 0.201) and 2-h glucose reduction (*p* < 0.001). Since only the first model increased the adjusted *R*^2^ (i.e., 64% to 68%), it was selected as the final model. Its prediction formula is as follows:

$$\ln\left(Hazard\;Ratio\right)=-1.79733+0.01037\times \mathrm{Mean baseline}\;2\;hour\;glucose\;\left[\mathrm{mg}/\mathrm{dL}\right]$$

(2) $$+0.02395\times 2\;hour\;glucose\;\mathrm{reduction}\;\left[\mathrm{mg}/\mathrm{dL}\right]$$

Table 2 shows the estimated risk reductions (%) for diabetes progression from the final model across the 24 hypothetical scenarios. When we set the baseline 2-h glucose at 140 mg/dL (7.8 mmol/L) and 2-h glucose reduction at 10 mg/dL (0.56 mmol/L), the corresponding risk reduction was 44%. When 2-h glucose reduction was increased to 40 mg/dL (2.2 mmol/L), the risk reduction rose to 73%. Furthermore, at any given 2-h glucose reduction, the prevention effect was observed to attenuate as baseline 2-h glucose increased. When 2-h glucose reduction was set at 10 mg/dL (0.56 mmol/L) for a baseline 2-h glucose of 190 mg/dL (10.6 mmol/L), the risk reduction was only 6%.

**Table 2:**
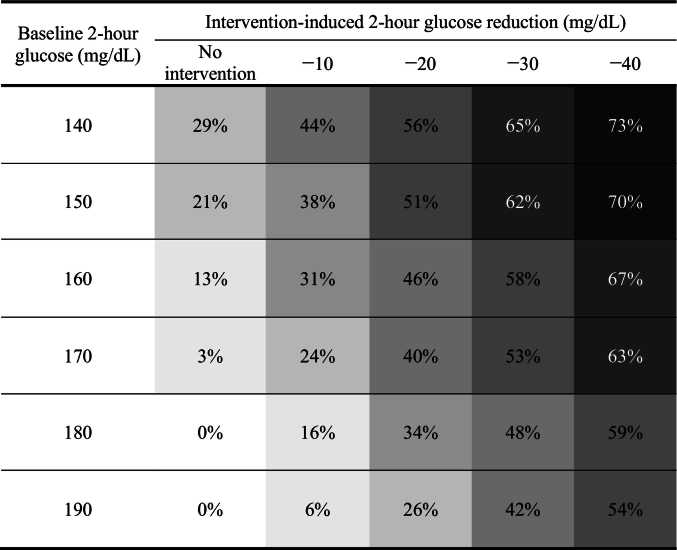
Estimated risk reduction for diabetes progression by 2-hour glucose change: meta-regression on change and baseline level of 2-hour glucose in type 2 diabetes prevention trials of adults with impaired glucose tolerance (1997–2024 Systematic Search).

Risk reductions (%) were calculated by incorporating the mean baseline 2-h glucose level and 2-h glucose reduction into the final meta-regression model.

A parallel meta-regression analysis using fasting glucose reduction and baseline fasting glucose met the criteria (both *p* < 0.2). The plot of log-transformed hazard ratio for diabetes progression against between-group differences in fasting glucose reduction was similar to that of the 2-h glucose model (**Supplementary Fig. S2**), and the estimated risk reductions were also generally comparable to those from the final model (**Supplementary Table S6**). However, the adjusted R-square indicated that the 2-h glucose model (68%) provided a better fit than the fasting glucose model (57%), which was even smaller than the univariate meta-regression with 2-h glucose reduction (64%).

To evaluate the influence of each study, we conducted a sensitivity analysis by sequentially excluding each of the 32 studies and reconstructing the meta-regression models (**Supplementary Table S7**). In all models, 2-h glucose reduction remained significantly associated with log-transformed hazard ratio for diabetes progression (*p* < 0.001), and baseline 2-h glucose consistently met the inclusion criterion of *p* < 0.2.

## 4 Discussion

We conducted a meta-regression analysis of randomized clinical trials to identify the optimal 2-h glucose reduction and baseline level for type 2 diabetes prevention in prediabetic individuals. The analysis enabled us to determine the optimal baseline 2-h glucose level at the intervention and to quantify the degree of 2-h glucose reduction required to prevent diabetes with a given risk reduction. A parallel meta-regression using fasting glucose parameters yielded similar trends, although the model fit was suboptimal.

Conventional lifestyle modifications, which represent first-line interventions, are expected to reduce 2-h glucose by a maximum of 10 mg/dL. As a result, such interventions would be effective in early-stage cases. In contrast, a larger 2-h glucose reduction of approximately 30 mg/dL (1.7 mmol/L) is needed to achieve comparable risk reductions in late-stage cases. Such reductions can be typically achieved through pharmacotherapy or more intensive lifestyle modifications. Pharmacotherapy can reduce 2-h glucose by up to 20–30 mg/dL (1.1–1.7 mmol/L), while intensive lifestyle modifications may induce even greater reductions of 40 mg/dL (2.2 mmol/L); however, these require a higher degree of patient commitment and adherence. Accordingly, these results underscore the importance of detecting early-stage impaired glucose tolerance to prevent diabetes progression.

There is substantial capacity for the restoration of normal glucose tolerance in the initial stages of impaired glucose tolerance, suggesting that the pathophysiological changes are still reversible (Warrilow et al., 2020). An analysis of data from 10 prospective European cohort studies identified impaired glucose tolerance as an independent risk factor for cardiovascular morbidity and mortality, highlighting its importance as a key target for early clinical intervention (DECODE Study Group, 2001). While prediabetes status is linked to increased cardiovascular risk (Huang et al., 2016), impaired glucose tolerance has demonstrated a higher propensity for progression to diabetes than impaired fasting glucose (Rao et al., 2004; Richter et al., 2018). In addition, studies have suggested that prediabetes interventions represent a cost-effective approach (Seuring et al., 2015; International Diabetes Federation, 2016; Pappachan and Ashraf, 2024). Although our findings suggest that pharmacotherapeutic treatments may be indicated for middle- or late-stage impaired glucose tolerance, their clinical benefits must be weighed against the increased risk of adverse events. Therefore, the implementation of minimal to moderate interventions (i.e., 2-h glucose reduction of 10–20 mg/dL or 0.56–1.1 mmol/L) during the early impaired glucose tolerance (i.e., 2-h glucose of 140–150 mg/dL or 7.8–8.3 mmol/L) represents the optimal strategy for diabetes prevention. By balancing strong preventive effects with reduced patient burden, this approach may be effective in halting the overall increase in incident diabetes.

To facilitate the early detection of impaired glucose tolerance, it is important to consider 2-h glucose in conjunction with fasting glucose. Previous studies have reported that fasting glucose alone failed to identify approximately 40–50% of impaired glucose tolerance (Eades et al., 2016) and 31% of diabetes (DECODE Study Group, 1999). Another study noted that 23% of hypertensive patients met the diagnostic criteria for impaired glucose tolerance despite having normal fasting glucose and hemoglobin A1c (Tugrul et al., 2009). Our model suggests that impaired glucose tolerance with a 2-h glucose of 170 mg/dL (9.4 mmol/L) or higher have an elevated risk of diabetes progression within several years if left untreated. Furthermore, research has indicated that 2-h glucose of 140 mg/dL (7.8 mmol/L) and 150 mg/dL (8.3 mmol/L) correspond to fasting glucose of 100 mg/dL (5.6 mmol/L) in persons aged <65 and ≥ 65 years, respectively (Ito et al., 2000). Routine assessment of postprandial glucose is warranted when fasting glucose exceed 100 mg/dL (5.6 mmol/L) to facilitate the early detection of impaired glucose tolerance. Moreover, a previous study reported that an fasting glucose within the high-normal range of 91–99 mg/dL (5.1–5.5 mmol/L) was a strong predictor of diabetes progression, and recommended implementing preventive measures (Nichols et al., 2008). A recent study has even proposed routine testing of 2-h glucose to ensure a more comprehensive risk assessment for diabetes (Lu et al., 2019).

The reproducibility of the 75-g oral glucose tolerance test is limited by day-to-day variability (Mooy et al., 1996; Ko et al., 1998; Libman et al., 2008), raising concerns about false positives. Although mitigating strategies such as repeated tests or combining hemoglobin A1c and fasting glucose have been proposed (American Diabetes Association Professional Practice Committee for Diabetes, 2026), repeated tolerance tests remain burdensome and hemoglobin A1c and fasting glucose frequently fail to detect impaired glucose tolerance and even diabetes. The tolerance test is the only method for identifying early impaired glucose tolerance, particularly isolated impaired glucose tolerance, which is more prevalent in Asian populations (Yip et al., 2017). However, its routine use in clinical practice poses practical challenges (The Expert Committee on the Diagnosis and Classification of Diabetes Mellitus, 1997).

This study has several limitations. First, since hemoglobin A1c changes were reported in only 17 of 32 studies, performing a meta-regression would yield unstable and potentially misleading estimates. Second, ecological bias may arise due to the use of aggregate data. Nevertheless, 2-h glucose remains one of the strongest predictive factors for diabetes (Noble et al., 2011; Nielsen et al., 2016). Across all of our models, the effects of 2-h glucose reduction were significant with a narrow 95% confidence band for the regression line. Third, some randomized clinical trials included participants with isolated impaired fasting glucose, which may have introduced confounding. We retained these studies because their populations predominantly comprised participants with impaired glucose tolerance; however, the potential influence of isolated impaired fasting glucose cases cannot be entirely excluded. Fourth, the intervention durations across studies ranged from 0.5 to 6 years, and the variability in intervention duration was not considered in the analysis. Therefore, differences in these durations may have influenced the results, and our findings should be interpreted with caution. Fifth, the meta-regression models only included intervenable variables as predictors, which introduces the risk of residual confounding. However, 2-h glucose reductions accounted for the majority of observed heterogeneity between the randomized clinical trials. Sixth, the study populations mainly consisted of individuals in their 40s and 50s at baseline, which could lead to selection bias. Nevertheless, impaired glucose tolerance typically develops in these age groups, and this demographic profile is unlikely to compromise the generalizability of our findings. Finally, there is the potential for publication bias, as small studies tended to lie below the regression line. However, the sensitivity analysis suggested that those studies did not have a large impact on the results.

## 5 Conclusions

Our meta-regression analysis demonstrated that both 2-h glucose reduction and baseline were significantly associated with diabetes progression in impaired glucose tolerance. Our results suggest that the required 2-h glucose reduction should be adjusted according to each patient's stage. Early-stage impaired glucose tolerance are more responsive to minimal interventions such as conventional lifestyle modifications, whereas middle- or late-stage cases may require pharmacotherapies or more intensive lifestyle modifications.

## Declaration of generative AI and AI-assisted technologies in the manuscript preparation process

During the preparation of this work, the authors used ChatGPT, Gemini, Claude and NotebookLM for comprehensive searching for useful literatures. After using these tools, the authors reviewed and edited the content as needed and take full responsibility for the content of the published article.

## CRediT authorship contribution statement

**Kotoba Okuyama:** Writing – review & editing, Writing – original draft, Visualization, Software, Methodology, Investigation, Formal analysis, Data curation, Conceptualization. **Anna Tsutsui:** Writing – review & editing, Validation, Supervision, Project administration, Conceptualization, Data curation, Investigation. **Yoshitaka Murakami:** Writing – review & editing, Supervision, Project administration, Conceptualization, Investigation.

## Funding

This research did not receive any specific grant from funding agencies in the public, commercial, or not-for-profit sectors.

## Declaration of competing interest

The authors declare that they have no known competing financial interests or personal relationships that could have appeared to influence the work reported in this paper.

## Data Availability

Table 1 includes all data which we analyzed.
