# Supplementary material for: Two-hour glucose reductions and baseline levels for effective prevention of type 2 diabetes in individuals with impaired glucose tolerance: An evidence synthesis and meta-regression analysis
Source: Prev Med Rep. 2026 Jul 13;69:103571. doi: 10.1016/j.pmedr.2026.103571 (PMC13400255; doi:10.1016/j.pmedr.2026.103571)
Supplement: Supplementary file 1 — Supplementary material. [file mmc1.docx]

Table S1 Search Expressions (PubMed)

| (lifestyle [Title] OR intervention [Title] OR modification [Title] OR diet [Title] OR nutrient [Title] OR supplement [Title] OR exercise [Title] OR physical [Title] OR anti-diabetic [Title] OR metformin [Title] OR acarbose [Title] OR voglibose [Title] OR glitazone [Title] OR rosiglitazone [Title] OR pioglitazone [Title] OR glinides [Title] OR repaglinide [Title] OR nateglinide [Title] OR glipizide [Title] OR glimepiride [Title] OR semaglutide [Title] OR sitagliptin [Title] OR liraglutide [Title] OR sulfonylurea* [Title] OR glinide* [Title] OR biguanide* [Title] OR "Alpha-glucosidase inhibitor" [Title] OR "DPP-4 inhibitor" [Title] OR "GLP-1 receptor agonist" [Title] OR "GLP-1" [Title] OR "glucagon like peptide" [Title] OR thiazolidine [Title] OR insulin [Title]) AND (Prediabetic state [Title] OR Glucose Intolerance [Title] OR prediabet* [Title] OR pre-diabet* [Title] OR glucose intolerance* [Title] OR glucose tolerance [Title] OR impaired glucose tolerance [Title] OR IGT [Title] OR diabetes [Title] OR T2D* [Title] OR NIDDM [Title] OR type 2* [Title] OR type II* [Title] OR dysglycemia [Title]) AND (effect* [Title] OR progress* [Title] OR prevent* [Title] OR inciden* [Title] OR conversion [Title] OR develop* [Title] OR delay* [Title] OR hazard* [tiab] OR long-term [Title] OR normalization [tiab]) NOT hypertensive [Title] NOT metabolic* [Title] NOT adiposity* [Title] NOT hypertension [Title] NOT web-based [Title] NOT questionnaire [Title] NOT biomarker* [Title] NOT pilot [Title] NOT pregnancy [Title] NOT gestational [Title] NOT hyperlipoproteinaemia [Title] NOT hyperlipidaemia [Title] NOT hypertriglyceridemia [Title] NOT hypercholesterolemia [Title] NOT young* [Title] NOT youth*[Title] NOT child* [Title] NOT renal* [Title] NOT liver* [Title] NOT cost* [Title] NOT pharmacoeconomic* [Title] NOT hypoglycemia [Title] NOT hour* [tiab] NOT 8-week* [tiab] NOT 16-week* [tiab] NOT 12-week* [tiab] NOT 16-week* [tiab] NOT 24-week* [tiab] NOT 6-month* [tiab] NOT dementia [Title] NOT crossover [Title] NOT cross-over [Title] NOT protocol [Title] NOT genetic* [Title] NOT type 1* [Title] NOT newly diagnosed type 2 diabe*[title] NOT with type 2 diabe* [title] NOT Diabetes Prevention Program [title] Filters: Full text, Randomized Controlled Trial, Humans, English, MEDLINE, Adult: 19-44 years, Middle Aged + Aged: 45+ years, Middle Aged: 45-64 years, Aged: 65+ years, 80 and over: 80+ years |
| --- |

Table S2 Search Expressions (Cochran library)

| ((([mh "Prediabetic State"] OR (Prediabetic State):ti,ab OR (prediabet*):ti,ab OR (pre-diabet*):ti,ab OR (Pre-DM):ti,ab) OR ([mh “Glucose Intolerance"] OR (glucose intolerance*):ti,ab OR (glucose tolerance):ti,ab OR (impaired glucose tolerance):ti,ab OR (IGT):ti,ab)) AND ([mh /TH] OR [mh /TU] OR (intervention*):ti,ab OR (modification*):ti,ab OR [mh therapeutics] OR (therapeutic*):ti,ab)) AND ([mh "Diabetes Mellitus, Type 2”/PC] OR (((type 2 diabetes mellitus):ti,ab OR (type II diabetes mellitus):ti,ab OR (Type 2 Diabetes):ti,ab OR (Type II Diabetes):ti,ab OR (T2D):ti,ab OR (T2DM):ti,ab OR (NIDDM):ti,ab OR (Noninsulin Dependent Diabetes Mellitus):ti,ab OR (dysglycemia):ti,ab OR (((Type 2):ti,ab OR (Type II):ti,ab) AND ((Diabetes):ti,ab OR (DM):ti,ab))) AND ((prevent*):ti,ab OR (intervent*):ti,ab OR ([mh prognosis] OR (prognosis):ti,ab OR (prognoses):ti,ab) OR (transmission*):ti,ab OR ((delay onset):ti,ab OR (onset*):ti,ab) OR (inciden*):ti,ab OR (develop*):ti,ab OR (conver*):ti,ab OR (treatment outcome):ti,ab OR [mh /PC] OR (therapeutic*):ti,ab OR [mh therapeutics]))) AND ((randomized controlled trial):ti,ab OR (controlled clinical trial):ti,ab OR (randomized):ti,ab OR (randomly):ti,ab OR (trial):ti,ab) NOT [mh animals] NOT ([mh child] OR [mh infant] OR [mh newborn] OR [mh child]) AND [mh humans] AND English:la AND (Journal article):pt |
| --- |

Limitations

with Complementary Medicine, Heart, Dementia and Cognitive Improvement, Effective Practice and Organisation of Care, Hypertension, Vascular, Stroke, Public Health, Musculoskeletal, Metabolic and Endocrine Disorders in Cochrane Groups

Table S3 Search Expressions (Ichu-shi library)

|  |
| --- |

Table S4 List of studies identified by systematic review

1. Pan XR, Li GW, Hu YH et al. Effects of diet and exercise in preventing NIDDM in people with impaired glucose tolerance. The Da Qing IGT and Diabetes Study. Diabetes Care. 1997 Apr;20(4):537-44. doi: 10.2337/diacare.20.4.537. PMID: 9096977.
2. Li CL, Pan CY, Lu JM et al. Effect of metformin on patients with impaired glucose tolerance. Diabet Med. 1999 Jun;16(6):477-81. doi: 10.1046/j.1464-5491.1999.00090.x. PMID: 10391395.
3. Tuomilehto J, Lindström J, Eriksson JG et al. Prevention of type 2 diabetes mellitus by changes in lifestyle among subjects with impaired glucose tolerance. N Engl J Med. 2001 May 3;344(18):1343-50. doi: 10.1056/NEJM200105033441801. PMID: 11333990.
4. Oldroyd JC, Unwin NC, White M et al. Randomised controlled trial evaluating lifestyle interventions in people with impaired glucose tolerance. Diabetes Res Clin Pract. 2006 May;72(2):117-27. doi: 10.1016/j.diabres.2005.09.018. Epub 2005 Nov 16. PMID: 16297488.
5. Eriksson JG, Lehtovirta M, Ehrnström B et al. Long-term beneficial effects of glipizide treatment on glucose tolerance in subjects with impaired glucose tolerance. J Intern Med. 2006 Jun;259(6):553-60. doi: 10.1111/j.1365-2796.2006.01633.x. PMID: 16704555.
6. Gerstein HC, Yusuf S, Bosch J et al. Effect of rosiglitazone on the frequency of diabetes in patients with impaired glucose tolerance or impaired fasting glucose: a randomised controlled trial. Lancet. 2006 Sep 23;368(9541):1096-105. doi: 10.1016/S0140-6736(06)69420-8. Erratum in: Lancet. 2006 Nov 18;368(9549):1770. PMID: 16997664.
7. Nijpels G, Boorsma W, Dekker JM et al. A study of the effects of acarbose on glucose metabolism in patients predisposed to developing diabetes: the Dutch acarbose intervention study in persons with impaired glucose tolerance (DAISI). Diabetes Metab Res Rev. 2008 Nov-Dec;24(8):611-6. doi: 10.1002/dmrr.839. PMID: 18756586.
8. Snehalatha C, Mary S, Selvam S et al. Changes in insulin secretion and insulin sensitivity in relation to the glycemic outcomes in subjects with impaired glucose tolerance in the Indian Diabetes Prevention Programme-1 (IDPP-1). Diabetes Care. 2009 Oct;32(10):1796-801. doi: 10.2337/dc09-0676. Epub 2009 Jul 8. PMID: 19587369; PMCID: PMC2752907.
9. Holman RR, Haffner SM, McMurray JJ et al, Effect of nateglinide on the incidence of diabetes and cardiovascular events. N Engl J Med. 2010 Apr 22;362(16):1463-76. doi: 10.1056/NEJMoa1001122. Epub 2010 Mar 14. Erratum in: N Engl J Med. 2010 May 6;362(18):1748. PMID: 20228402.
10. DeFronzo RA, Tripathy D, Schwenke DC et al. ACT NOW Study. Pioglitazone for diabetes prevention in impaired glucose tolerance. N Engl J Med. 2011 Mar 24;364(12):1104-15. doi: 10.1056/NEJMoa1010949. Erratum in: N Engl J Med. 2011 Jul 14;365(2):189. Erratum in: N Engl J Med. 2011 Sep 1;365(9):869. PMID: 21428766.
11. Roumen C, Feskens EJ, Corpeleijn E et al. Predictors of lifestyle intervention outcome and dropout: the SLIM study. Eur J Clin Nutr. 2011 Oct;65(10):1141-7. doi: 10.1038/ejcn.2011.74. Epub 2011 May 18. PMID: 21587283.
12. Sakane N, Sato J, Tsushita K et al. Japan Diabetes Prevention Program (JDPP) Research Group. Prevention of type 2 diabetes in a primary healthcare setting: three-year results of lifestyle intervention in Japanese subjects with impaired glucose tolerance. BMC Public Health. 2011 Jan 17;11(1):40. doi: 10.1186/1471-2458-11-40. PMID: 21235825; PMCID: PMC3037863.
13. Saito T, Watanabe M, Nishida J et al. Zensharen Study for Prevention of Lifestyle Diseases Group. Lifestyle modification and prevention of type 2 diabetes in overweight Japanese with impaired fasting glucose levels: a randomized controlled trial. Arch Intern Med. 2011 Aug 8;171(15):1352-60. doi: 10.1001/archinternmed.2011.275. PMID: 21824948.
14. Moore SM, Hardie EA, Hackworth NJ et al. Can the onset of type 2 diabetes be delayed by a group-based lifestyle intervention? A randomised control trial. Psychol Health. 2011 Apr;26(4):485-99. doi: 10.1080/08870440903548749. Epub 2010 Oct 11. PMID: 20945253.
15. Monti LD, Setola E, Lucotti PC et al. Effect of a long-term oral l-arginine supplementation on glucose metabolism: a randomized, double-blind, placebo-controlled trial. Diabetes Obes Metab. 2012 Oct;14(10):893-900. doi: 10.1111/j.1463-1326.2012.01615.x. Epub 2012 May 21. PMID: 22553931.
16. Gao Y, Zhou H, Zhao H et al. Clinical research of traditional Chinese medical intervention on impaired glucose tolerance. Am J Chin Med. 2013;41(1):21-32. doi: 10.1142/S0192415X1350002X. PMID: 23336504.
17. Wong CK, Fung CS, Siu SC et al. A short message service (SMS) intervention to prevent diabetes in Chinese professional drivers with pre-diabetes: a pilot single-blinded randomized controlled trial. Diabetes Res Clin Pract. 2013 Dec;102(3):158-66. doi: 10.1016/j.diabres.2013.10.002. PMID: 24466598.
18. Dutta D, Mondal SA, Choudhuri S et al. Vitamin-D supplementation in prediabetes reduced progression to type 2 diabetes and was associated with decreased insulin resistance and systemic inflammation: an open label randomized prospective study from Eastern India. Diabetes Res Clin Pract. 2014 Mar;103(3):e18-23. doi: 10.1016/j.diabres.2013.12.044. Epub 2014 Jan 6. PMID: 24456991.
19. Gaddam A, Galla C, Thummisetti S et al. Role of Fenugreek in the prevention of type 2 diabetes mellitus in prediabetes. J Diabetes Metab Disord. 2015 Oct 2;14:74. doi: 10.1186/s40200-015-0208-4. PMID: 26436069; PMCID: PMC4591578.
20. Jorde R, Sollid ST, Svartberg J et al. Vitamin D 20,000 IU per Week for Five Years Does Not Prevent Progression From Prediabetes to Diabetes. J Clin Endocrinol Metab. 2016 Apr;101(4):1647-55. doi: 10.1210/jc.2015-4013. Epub 2016 Feb 1. PMID: 26829443.
21. Holman RR, Coleman RL, Chan JCN et al. Effects of acarbose on cardiovascular and diabetes outcomes in patients with coronary heart disease and impaired glucose tolerance (ACE): a randomised, double-blind, placebo-controlled trial. Lancet Diabetes Endocrinol. 2017 Nov;5(11):877-886. doi: 10.1016/S2213-8587(17)30309-1. Epub 2017 Sep 13. Erratum in: Lancet Diabetes Endocrinol. 2017 Nov;5(11):e7. doi: 10.1016/S2213-8587(17)30335-2. Erratum in: Lancet Diabetes Endocrinol. 2019 May;7(5):e5. doi: 10.1016/S2213-8587(19)30063-4. PMID: 28917545.
22. le Roux CW, Astrup A, Fujioka K et al. 3 years of liraglutide versus placebo for type 2 diabetes risk reduction and weight management in individuals with prediabetes: a randomised, double-blind trial. Lancet. 2017 Apr 8;389(10077):1399-1409. doi: 10.1016/S0140-6736(17)30069-7. Epub 2017 Feb 23. Erratum in: Lancet. 2017 Apr 8;389(10077):1398. doi: 10.1016/S0140-6736(17)30705-5. PMID: 28237263.
23. Hu Z, Qin L, Xu H. One-Year Results of a Synthetic Intervention Model for the Primary Prevention of T2D among Elderly Individuals with Prediabetes in Rural China. Int J Environ Res Public Health. 2017 Apr 14;14(4):417. doi: 10.3390/ijerph14040417. PMID: 28420105; PMCID: PMC5409618.
24. Honsek C, Kabisch S, Kemper M et al. Fibre supplementation for the prevention of type 2 diabetes and improvement of glucose metabolism: the randomised controlled Optimal Fibre Trial (OptiFiT). Diabetologia. 2018 Jun;61(6):1295-1305. doi: 10.1007/s00125-018-4582-6. Epub 2018 Feb 28. PMID: 29492637.
25. Ranasinghe P, Wathurapatha WS, Galappatthy P et al. Zinc supplementation in prediabetes: A randomized double-blind placebo-controlled clinical trial. J Diabetes. 2018 May;10(5):386-397. doi: 10.1111/1753-0407.12621. Epub 2018 Jan 3. PMID: 29072815.
26. Niroomand M, Fotouhi A, Irannejad N et al. Does high-dose vitamin D supplementation impact insulin resistance and risk of development of diabetes in patients with pre-diabetes? A double-blind randomized clinical trial. Diabetes Res Clin Pract. 2019 Feb;148:1-9. doi: 10.1016/j.diabres.2018.12.008. Epub 2018 Dec 21. PMID: 30583032.
27. Nakanekar A, Kohli K, Tatke P. Ayurvedic polyherbal combination (PDBT) for prediabetes: A randomized double blind placebo controlled study. J Ayurveda Integr Med. 2019 Oct-Dec;10(4):284-289. doi: 10.1016/j.jaim.2018.05.004. Epub 2019 Jan 17. PMID: 30661947; PMCID: PMC6938901.
28. Guardado-Mendoza R, Salazar-López SS, Álvarez-Canales M et al. The combination of linagliptin, metformin and lifestyle modification to prevent type 2 diabetes (PRELLIM). A randomized clinical trial. Metabolism. 2020 Mar;104:154054. doi: 10.1016/j.metabol.2019.154054. Epub 2019 Dec 28. PMID: 31887309.
29. Raben A, Vestentoft PS, Brand-Miller J et al. The PREVIEW intervention study: Results from a 3-year randomized 2 x 2 factorial multinational trial investigating the role of protein, glycaemic index and physical activity for prevention of type 2 diabetes. Diabetes Obes Metab. 2021 Feb;23(2):324-337. doi: 10.1111/dom.14219. Epub 2020 Nov 3. PMID: 33026154; PMCID: PMC8120810.
30. Bhatt SP, Misra A, Pandey RM　et al. Vitamin D Supplementation in Overweight/obese Asian Indian Women with Prediabetes Reduces Glycemic Measures and Truncal Subcutaneous Fat: A 78 Weeks Randomized Placebo-Controlled Trial (PREVENT-WIN Trial). Sci Rep. 2020 Jan 14;10(1):220. doi: 10.1038/s41598-019-56904-y. Erratum in: Sci Rep. 2020 Jun 12;10(1):9844. doi: 10.1038/s41598-020-67064-9. PMID: 31937856; PMCID: PMC6959323.
31. Ji H, Zhao X, Chen X et al. Jinlida for Diabetes Prevention in Impaired Glucose Tolerance and Multiple Metabolic Abnormalities: The FOCUS Randomized Clinical Trial. JAMA Intern Med. 2024 Jul 1;184(7):727-735. doi: 10.1001/jamainternmed.2024.1190. Erratum in: JAMA Intern Med. 2024 Sep 1;184(9):1137. doi: 10.1001/jamainternmed.2024.3409. Erratum in: JAMA Intern Med. 2024 Oct 1;184(10):1270. doi: 10.1001/jamainternmed.2024.4669. PMID: 38829648; PMCID: PMC11148787.
32. Madhu SV, Rao PV, Chandalia HB et al. Yoga and prevention of type 2 diabetes - The Indian Prevention of Diabetes Study (IPDS). Diabetes Metab Syndr. 2024 Jul;18(7):103088. doi: 10.1016/j.dsx.2024.103088. Epub 2024 Jul 27. PMID: 39079307.

Table S5 Variable selection in the meta-regression analysis by 2-hour glucose reduction in type 2 diabetes prevention trials of adults with impaired glucose tolerance (1997–2024 systematic search)

| **Number of Studies** | **P-values** | | | | **Adjusted R-square (%)** |
| --- | --- | --- | --- | --- | --- |
|  | **Two-hour glucose reduction (vs control)** | **Two-hour glucose**  **at baseline** | **Fasting glucose**  **at baseline** | **Body Mass Index**  **at baseline** |  |
| 31 | <0.001 | 0.102 | 0.540 | 0.335 | 67 |
| 32 | <0.001 | 0.045 | 0.939 | . | 67 |
| 31 | <0.001 | 0.088 | . | 0.445 | 68 |
| 31 | <0.001 | . | 0.468 | 0.149 | 65 |
| **32** | **<0.001** | **0.041** | **.** | **.** | **68** |
| 32 | <0.001 | . | 0.897 | . | 63 |
| 31 | <0.001 | . | . | 0.201 | 65 |

Table S6 Estimated risk reduction for diabetes progression by fasting glucose reduction: meta-regression on change and baseline level of fasting glucose in type 2 diabetes prevention trials of adults with impaired glucose tolerance (1997–2024 systematic search)

| **Baseline fasting glucose**  **(mg/dL)** | **Two-hour glucose (mg/dL)** ^†^ | **Two-hour glucose (mg/dL) ^‡^** | **Intervention-induced fasting glucose reduction (mg/dL)** | | | | |
| --- | --- | --- | --- | --- | --- | --- | --- |
|  |  |  | **No**  **intervention** | **−5** | **−10** | **−15** | **−20** |
| 100 | 141 | 151 | 36% | 51% | 62% | 71% | 78% |
| 102 | 146 | 155 | 31% | 47% | 59% | 68% | 76% |
| 104 | 150 | 160 | 25% | 42% | 55% | 66% | 74% |
| 106 | 155 | 164 | 18% | 37% | 52% | 63% | 71% |
| 108 | 159 | 169 | 11% | 32% | 47% | 60% | 69% |
| 110 | 164 | 174 | 4% | 26% | 43% | 56% | 66% |
| 112 | 168 | 178 | 0% | 20% | 38% | 52% | 63% |
| 114 | 173 | 183 | 0% | 13% | 33% | 48% | 60% |
| 116 | 177 | 188 | 0% | 6% | 27% | 44% | 57% |
| 118 | 182 | 192 | 0% | 0% | 21% | 39% | 53% |
| 120 | 186 | 197 | 0% | 0% | 14% | 34% | 49% |
| 122 | 191 | 201 | 0% | 0% | 7% | 28% | 45% |
| 124 | 195 | 206 | 0% | 0% | 0% | 22% | 40% |
| 126 | 200 | 211 | 0% | 0% | 0% | 16% | 35% |

Studies from Table 1 that reported 2-hour glucose data were included in this meta-regression. ^†^ Two-hour glucose = −82.1+2.235*Fasting glucose (mg/dL) (<60 yrs); ^‡^ Two-hour glucose = −81.1+2.316*Fasting glucose (mg/dL) (≥60 yrs) [Ito, et al, 2000]

Risk reductions (%) were calculated by incorporating the mean baseline fasting glucose level and fasting glucose reduction into the meta-regression model (n=29).

Table S7 Sensitivity analysis evaluating the influence of individual studies on the final meta-regression model with 2-hour glucose parameters by excluding studies one-by-one: studies of type 2 diabetes prevention of adults with impaired glucose tolerance (1997–2024 systematic search)

| **Study No. Excluding from**  **meta-regression model** | **Two-hour glucose reduction (vs control)** | | **Mean Two-hour glucose at baseline** | | **Adjusted *R*^2^ square (%)** | **Hazard Ratio** | **Risk Reduction**  **(%)** |
| --- | --- | --- | --- | --- | --- | --- | --- |
|  | **Regression coefficient** | **P-value** | **Regression coefficient** | **P-value** |  |  |  |
| **All studies included** | **0.024** | **<.001** | **0.010** | **0.041** | **68** | **0.47** | **53** |
| 1 | 0.027 | <.001 | 0.009 | 0.052 | 73 | 0.42 | 58 |
| 2 | 0.024 | <.001 | 0.010 | 0.044 | 68 | 0.47 | 53 |
| 3 | 0.024 | <.001 | 0.010 | 0.042 | 69 | 0.47 | 53 |
| 4 | 0.024 | <.001 | 0.010 | 0.044 | 68 | 0.47 | 53 |
| 5 | 0.024 | <.001 | 0.010 | 0.046 | 68 | 0.47 | 53 |
| 6 | 0.022 | <.001 | 0.010 | 0.049 | 54 | 0.51 | 49 |
| 7 | 0.024 | <.001 | 0.010 | 0.048 | 68 | 0.47 | 53 |
| 8 | 0.024 | <.001 | 0.011 | 0.035 | 69 | 0.47 | 53 |
| 9 | 0.019 | <.001 | 0.007 | 0.173 | 35 | 0.47 | 53 |
| 10 | 0.024 | <.001 | 0.011 | 0.026 | 70 | 0.48 | 52 |
| 11 | 0.024 | <.001 | 0.010 | 0.045 | 68 | 0.47 | 53 |
| 12 | 0.024 | <.001 | 0.010 | 0.042 | 68 | 0.47 | 53 |
| 13 | 0.024 | <.001 | 0.010 | 0.057 | 68 | 0.47 | 53 |
| 14 | 0.024 | <.001 | 0.011 | 0.028 | 71 | 0.47 | 53 |
| 15 | 0.024 | <.001 | 0.010 | 0.044 | 68 | 0.47 | 53 |
| 16 | 0.026 | <.001 | 0.009 | 0.081 | 69 | 0.44 | 56 |
| 17 | 0.024 | <.001 | 0.011 | 0.043 | 68 | 0.47 | 53 |
| 18 | 0.024 | <.001 | 0.010 | 0.045 | 68 | 0.47 | 53 |
| 19 | 0.024 | <.001 | 0.010 | 0.055 | 68 | 0.47 | 53 |
| 20 | 0.022 | <.001 | 0.018 | 0.006 | 72 | 0.52 | 48 |
| 21 | 0.024 | <.001 | 0.010 | 0.050 | 68 | 0.47 | 53 |
| 22 | 0.024 | <.001 | 0.008 | 0.111 | 67 | 0.47 | 53 |
| 23 | 0.024 | <.001 | 0.010 | 0.039 | 72 | 0.47 | 53 |
| 24 | 0.024 | <.001 | 0.010 | 0.043 | 68 | 0.47 | 53 |
| 25 | 0.024 | <.001 | 0.010 | 0.043 | 68 | 0.47 | 53 |
| 26 | 0.024 | <.001 | 0.010 | 0.047 | 68 | 0.47 | 53 |
| 27 | 0.024 | <.001 | 0.010 | 0.045 | 68 | 0.47 | 53 |
| 28 | 0.024 | <.001 | 0.010 | 0.040 | 69 | 0.47 | 53 |
| 29 | 0.024 | <.001 | 0.011 | 0.043 | 68 | 0.47 | 53 |
| 30 | 0.024 | <.001 | 0.010 | 0.047 | 68 | 0.47 | 53 |
| 31 | 0.024 | <.001 | 0.011 | 0.033 | 69 | 0.48 | 52 |
| 32 | 0.024 | <.001 | 0.009 | 0.068 | 69 | 0.47 | 53 |
| † Hazard ratio and risk reduction [(1 minus hazard ratio)x100%] were calculated under the specific condition which mean two-hour glucose at baseline and in two-hour glucose reduction (vs control) were set as 170 mg/dL and -30 mg/dL, respectively. | | | | | | | |

Figure S1 Scatter plot comparing estimated and calculated hazard ratios for type 2 diabetes progression among studies reported the hazard ratios: studies of type 2 diabetes prevention of adults with impaired glucose tolerance (1997–2024 systematic search)


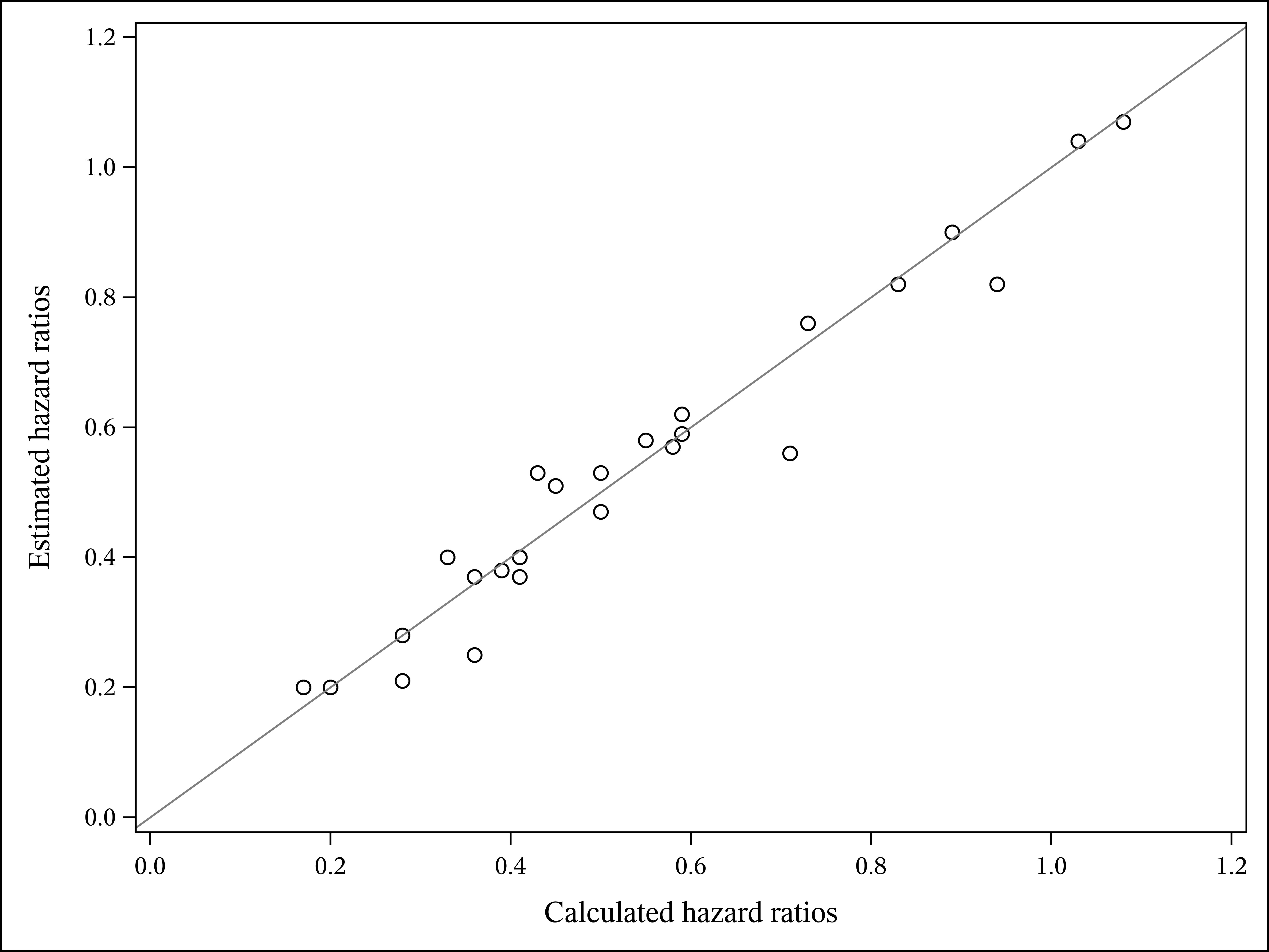


Solid line: 45-degree reference line

Figure S2. Univariate meta-regression of log hazard ratios for type 2 diabetes progression by fasting glucose reduction (vs. control) in type 2 diabetes prevention trials of adults with impaired glucose tolerance reporting 2-hour glucose data (1997–2024 systematic search)


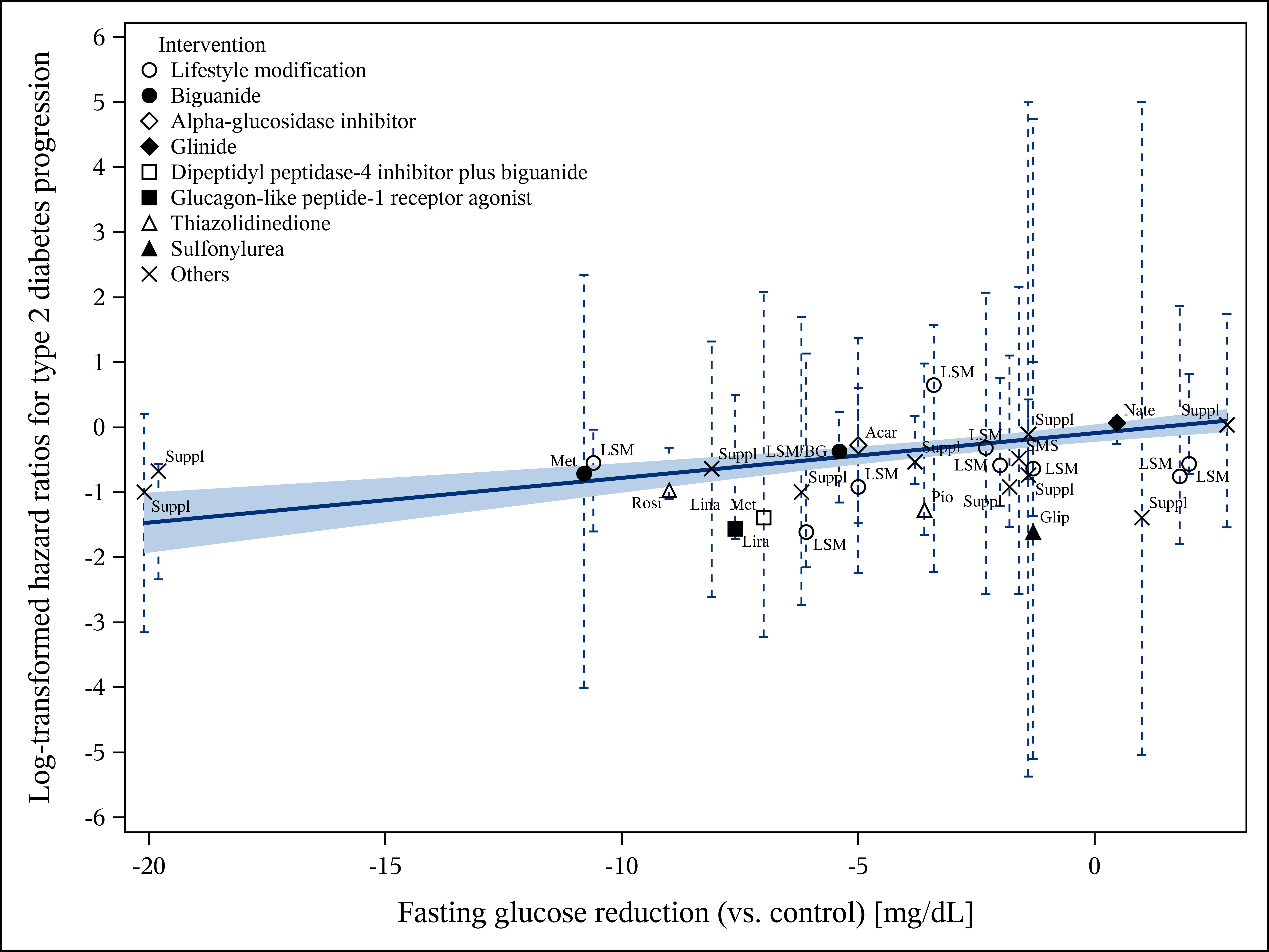


The solid blue line indicates the predicted meta-regression line, and the shaded blue area represents its 95% confidence band. The dashed vertical lines indicate the predicted 95% confidence intervals for individual predicted values in each study. “Other” refers to Chinese medicines, supplements (e.g., vitamin D and calcium), and SMS.

Abbreviations: Acar, acarbose; AGI, alpha-glucosidase inhibitor; DPP4, dipeptidyl peptidase-4 inhibitor; Glip, glipizide; GLP-1, glucagon-like peptide-1 receptor agonist; Lina, linagliptin; Lira, liraglutide; LSM, lifestyle modification; Met, metformin; Nate, nateglinide; Pio, pioglitazone; Rosi, rosiglitazone; SMS, Short Message Service; Suppl, supplements; TZD, thiazolidinedione.
